# Strong optomechanical coupling at room temperature by coherent scattering

**DOI:** 10.1038/s41467-020-20419-2

**Published:** 2021-01-12

**Authors:** Andrés de los Ríos Sommer, Nadine Meyer, Romain Quidant

**Affiliations:** 1 ICFO-Institut de Ciencies Fotoniques, The Barcelona Institute of Science and Technology, 08860 Castelldefels (Barcelona), Spain; 2 ICREA-Institució Catalana de Recerca i Estudis Avançats, 08010 Barcelona, Spain; 3 Nanophotonic Systems Laboratory, Department of Mechanical and Process Engineering, ETH Zurich, 8092 Zurich, Switzerland

**Keywords:** Nanoparticles, Optical manipulation and tweezers, Optical physics

## Abstract

Quantum control of a system requires the manipulation of quantum states faster than any decoherence rate. For mesoscopic systems, this has so far only been reached by few cryogenic systems. An important milestone towards quantum control is the so-called strong coupling regime, which in cavity optomechanics corresponds to an optomechanical coupling strength larger than cavity decay rate and mechanical damping. Here, we demonstrate the strong coupling regime at room temperature between a levitated silica particle and a high finesse optical cavity. Normal mode splitting is achieved by employing coherent scattering, instead of directly driving the cavity. The coupling strength achieved here approaches three times the cavity linewidth, crossing deep into the strong coupling regime. Entering the strong coupling regime is an essential step towards quantum control with mesoscopic objects at room temperature.

## Abstract (web summary)

Reaching the strong coupling regime is a crucial step towards room-temperature quantum control with mesoscopic objects. Here, the authors use coherent scattering to demonstrate room temperature strong coupling between a levitated silica particle and a high-finesse optical cavity.

## Introduction

Laser cooling has revolutionised our understanding of atoms, ions and molecules. Lately, after a decade of experimental and theoretical efforts employing the same techniques^1–8^, the motional ground state of levitated silica nanoparticles at room temperature has been reported^9^. While this represents an important milestone towards the creation of mesoscopic quantum objects, coherent quantum control of levitated nanoparticles^10,11^ still remains elusive.

Levitated particles stand out among the plethora of optomechanical systems^12^ due to their detachment, and therefore high degree of isolation from the environment. Their centre of mass, rotational and vibrational degrees of freedom^13^ make them attractive tools for inertial sensing^14^, rotational dynamics^15–18^, free fall experiments^19^, exploration of dynamic potentials^20^, and are envisioned for testing macroscopic quantum phenomena at room temperature^2,10,21,22^.

Recently, the centre-of-mass motion of a levitated particle has successfully been 3D cooled employing coherent scattering (CS)^8,23^. Cooling with CS is less sensitive to phase noise heating than actively driving the cavity^7,24^, because optimal coupling takes place at the intensity node. Lately, this has enabled phonon occupation numbers of less than one^9^.

For controlled quantum experiments, such as the preparation of non-classical, squeezed^25,26^ or entangled states^27,28^, the particle’s motional state needs to be manipulated faster than the absorption of a single phonon from the environment. A valuable but less stringent condition is the so-called strong coupling regime (SCR), where the optomechanical coupling strength *g* between the mechanical motion of a particle and an external optical cavity exceeds the particle’s mechanical damping Γ_m_ and the cavity linewidth *κ* (*g* ≫ Γ_m_, *κ*). The SCR presents one of the first stepping stones towards full quantum control and has been demonstrated in opto- and electromechanical systems^29–31^, followed by quantum-coherent control^32^.

Here, we observe normal mode splitting (NMS) in SCR with levitated nanoparticles^33^, as originally reported in atoms^34^. In contrast to previous experiments, we employ CS^8,23,35,36^. Our table-top experiment offers numerous ways to tune the optomechanical coupling strength at room temperature, a working regime that is otherwise nearly exclusive to plasmonic nanocavities^37,38^.

## Results

### Experimental setup for levitation

**Fig. 1:**
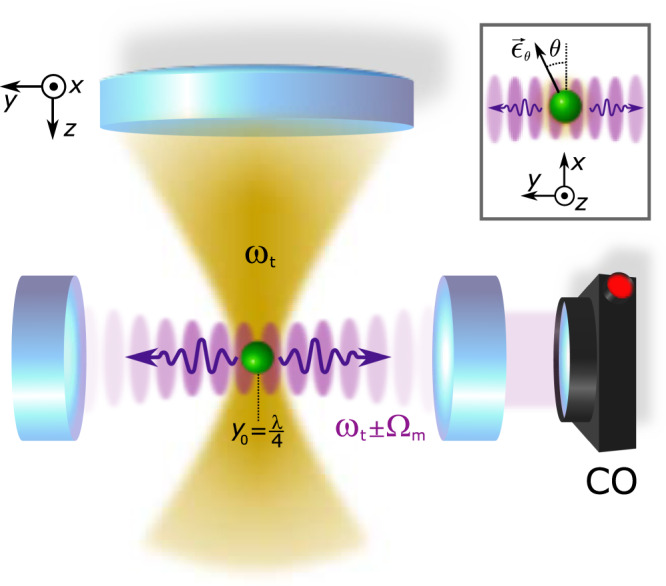
Experimental setup. An optical tweezers trap (yellow) levitates a silica nanoparticle inside a high finesse cavity. The trapping field is locked relatively to the cavity resonance *ω*_cav_ using Pound-Drever-Hall locking with a detuning Δ = *ω*_t_ − *ω*_cav_. A 3D piezo stage positions the particle precisely inside the cavity at variable *y*_0_. The inset displays the linear trap polarisation axis along ${\epsilon }_{\theta }={\epsilon }_{{{x}}}\cos \theta$. The rate of coherently scattered photons into the cavity mode (purple) depends on *y*_0_, *ϵ*_*θ*_ and Δ. The transmitted cavity output field is monitored on a photodiode (CO) and the forward scattered trapping light is used to detect the particle motion (see “Experimental setup” section).

Our experimental setup is displayed in Fig. 1. A silica nanoparticle (green) of radius *R* ≈ 90 nm, mass *m* = 6.4 × 10^−18^ kg and refractive index *n*_r_ = 1.45 is placed in a cavity (purple) by an optical tweezers trap (yellow) with wavelength $\lambda$_t_ = 2*π*/*k*_t_ = 1064 nm, power *P*_t_ ≃ 150 mW, numerical aperture NA = 0.8, and optical axis (*z*) perpendicular to the cavity axis (*y*). The trap is linearly polarised along the axis defined as ${\epsilon }_{\theta }={\epsilon }_{{{x}}}\cos \theta$ (see inset in Fig. 1).

The nanoparticle’s eigenfrequencies Ω_*x*,*y*,*z*_ = 2*π* × (172 kHz, 197 kHz, 56 kHz) are non-degenerate due to tight focusing. The trap is mounted on a nano-positioning stage allowing for precise 3D placement of the particle inside the low loss, high finesse Fabry-Pérot cavity with a cavity linewidth *κ* ≈ 2*π* × 10 kHz, cavity finesse *F* = 5.4 × 10^5^ and free spectral range Δ*ω*_FSR_ = *πc*/*L*_c_ = 2*π* × 5.4 GHz. The relative detuning Δ = *ω*_t_ − *ω*_c_ between the trap and the cavity resonance is tunable. The intracavity photon number *n*_cav_ is estimated from the transmitted cavity power *P*_out_ (CO in Fig. 1), and the particle position displacement is measured by interfering the scattered light with a co-propagating reference beam^39^. In CS, scattering events from the detuned trapping field, locked at Δ, populate the cavity. This contrasts the approach of actively driving the cavity^3,7,24^. A particle in free space, solely interacting with the trapping light, Raman scatters photons into free space and the energy difference between incident and emitted light equals ±*ℏ*Ω_*m*_ with *m* = *x*, *y*, *z*. In this case, photon up and down conversion are equally probable^40^. The presence of an optical cavity alters the density of states of electromagnetic modes and enhances the CS into the cavity modes through the Purcell effect. If trap photons are red (blue) detuned with respect to the cavity resonance, the cavity enhances photon up (down) conversion and net cooling (heating) takes place.

### Coherent scattering theory

In order to estimate the corresponding optomechanical coupling strength in CS, we follow ref. ^36^. The interaction Hamiltonian for a polarisable particle interacting with an electric field **E**(**R**) is given by ${\hat{{\rm{H}}}}_{{\rm{int}}}=-\frac{1}{2}\alpha {{\bf{E}}}^{2}({\bf{R}})$ with the particle polarizability $\alpha =4\pi {\epsilon }_{0}{R}^{3}\frac{{n}_{{\rm{r}}}^{2}-1}{{n}_{{\rm{r}}}^{2}+2}$ and vacuum permittivity *ϵ*_0_. The total electric field consists of the trap (${{\bf{E}}}_{{\rm{tr}}}({\bf{R}})$), cavity (**E**_cav_(**R**)) and free space electromagnetic modes (**E**_free_(**R**)) yielding the interaction Hamiltonian

1 $${\hat{{\rm{H}}}}_{{\rm{int}}}=-\frac{1}{2}\alpha {\left[{{\bf{E}}}_{{\rm{tr}}}({\bf{R}})+{{\bf{E}}}_{{\rm{cav}}}({\bf{R}})+{{\bf{E}}}_{{\rm{free}}}({\bf{R}})\right]}^{2}$$

2 $$\approx {\hat{{\rm{H}}}}_{{\rm{CS}}}+{\hat{{\rm{H}}}}_{{\rm{DR}}}+{\hat{{\rm{H}}}}_{{\rm{CAV}}}$$

where **E**_cav_(**R**) and **E**_free_(**R**) are only populated by scattering events from the particle (*n*_trap_ ≫ *n*_cav_ with *n*_trap_ (*n*_cav_) being the number of trap (cavity) photons). As can be seen from Eq. (1), the interaction Hamiltonian consists of six terms of which only the two terms proportional to ${{\bf{E}}}_{{\rm{tr}}}({\bf{R}}){{\bf{E}}}_{{\rm{cav}}}({\bf{R}})$ and **E**_cav_(**R**)^2^ are relevant for the following discussion^36^. The former one gives rise to the optomechanical coupling by CS, and the latter to the coupling achieved by actively driving the cavity. The term $\propto {{\bf{E}}}_{{\rm{tr}}}^{2}({\bf{R}})$ gives rise to the trapping potential, while the term $\propto {{\bf{E}}}_{{\rm{tr}}}({\bf{R}}){{\bf{E}}}_{{\rm{free}}}({\bf{R}})$ causes recoil heating^36,41^, which can be neglected for the moderate vacuum conditions presented here^7,41^. The remaining two terms can be safely neglected according to ref. ^36^.

In the following, we use the simplified interaction Hamiltonian given by Eq. (2) where we separate the parts contributing to the optomechanical coupling due to CS ${\hat{{\rm{H}}}}_{{\rm{CS}}}$, active driving ${\hat{{\rm{H}}}}_{{\rm{DR}}}$, and population of the intracavity field ${\hat{{\rm{H}}}}_{{\rm{CAV}}}$ (see “Interaction Hamiltonian and power spectral densities” section).

For the measurements presented here, the trap is *x*-polarised with *θ* = 0 (see inset Fig. 1). This simplifies ${\hat{{\rm{H}}}}_{{\rm{CS}}}$ to ${\hat{{\rm{H}}}}_{{\rm{CS}}}=-\hslash [{g}_{{{y}}}({\hat{a}}^{\dagger }+\hat{a})({\hat{{b}_{y}}}^{\dagger }+\hat{{b}_{y}})+{g}_{{{z}}}({\hat{a}}^{\dagger }-\hat{a})({\hat{{b}_{z}}}^{\dagger }+\hat{{b}_{z}})]$, where $\hat{a}$ (${\hat{a}}^{\dagger }$) is the photon annihilation (creation) operator and $\hat{b}$ (${\hat{b}}^{\dagger }$) is the phonon annihilation (creation) operator. The CS optomechanical coupling strengths *g*_*y*,*z*_ are

3 $$\left[\begin{array}{c}{g}_{y}\\ {g}_{z}\end{array}\right]=\frac{1}{2}\left[\begin{array}{r}{G}_{\perp }{k}_{{\rm{c}}}{y}_{{\rm{zpf}}}\sin \phi \\ -i\,\,{G}_{\perp }{k}_{{\rm{t}}}{z}_{{\rm{zpf}}}\cos \phi \end{array}\right]$$

with cavity wavevector *k*_c_ = 2*π*/$\lambda$_c_, zero-point fluctuations ${y}_{{\rm{zpf}}},{z}_{{\rm{zpf}}}=\sqrt{\frac{\hslash }{2m{{{\Omega }}}_{{{y}},{{z}}}}}$ and *ϕ* = 2*πy*_0_/$\lambda$_c_, with *y*_0_ being the particle position along the cavity axis and *y*_0_ = $\lambda$_c_/4 corresponding to the intensity minimum.

The optical cavity resonance frequency shift caused by a particle located at maximum intensity of the intracavity standing wave is ${G}_{\perp }=\alpha {E}_{0}\sqrt{\frac{{\omega }_{{\rm{c}}}}{2\hslash {\epsilon }_{0}{V}_{{\rm{c}}}}}$ with cavity mode volume ${V}_{{\rm{c}}}=\pi {{\rm{w}}}_{{\rm{c}}}^{2}{L}_{{\rm{c}}}/4$, cavity waist *w*_c_, cavity length *L*_c_, and *ω*_c_ = 2*πc*/$\lambda$_c_ The trap electric field is ${E}_{0}=\sqrt{\frac{4{P}_{{\rm{t}}}}{\pi {\epsilon }_{0}c{{{\mathrm{w}}}}_{{{x}}}{{{\mathrm{w}}}}_{{{y}}}}}$ with trap waists *w*_*x*_ and *w*_*y*_.

Due to the intracavity standing wave, the optomechanical coupling strength has a sinusoidal dependence on *y*_0_ with opposite phase for *g*_*y*_ and *g*_*z*_. In contrast, *g*_*x*_ = 0 if *θ* = 0.

For clarity, we limit the discussion to coupling along the cavity axis (*y*), such that Ω_*m*_ = Ω_*y*_ and *g* = *g*_*y*_. Similar results can be obtained for the other directions *x*, *z* with the same level of control.

The maximum expected coupling strength from CS is ${g}_{{{y}}}^{\max }={G}_{\perp }{k}_{{\rm{c}}}\,{y}_{{\rm{zpf}}}=2\pi \times 31.7\ {\rm{kHz}}$ for our parameters. However, we displace the particle by *δz* ≈ 40 μm from the cavity centre for better experimental stability. Hence, our expected optomechanical coupling strength is reduced by ≈30% down to ${g}_{{{y}}}^{{\rm{th}}}=2\pi \times 22.6\ {\rm{kHz}}$, enabling the SCR with *g*_*y*_ > *κ*. Despite the fact that this value is a factor of ≈3 lower than previously reported^9^, the deep SRC with *g* > *κ* remains unaccomplished.

### Transition to the SCR

In the weak coupling regime *g* < *κ*, the Lorentzian-shaped spectra of our mechanical oscillator displays a single peak at its resonance frequency Ω_*m*_. When *g* increases, the energy exchange rate between optical and mechanical mode grows until the SCR is reached at *g* > *κ*/4 (ref. ^33^). In the SCR, the optical and mechanical mode hybridise, which gives rise to two new eigenmodes at shifted eigenfrequencies Ω_±_ (see Eq. (9)). At this point, the energy exchange in between the optical and mechanical mode is faster than the decoherence rate of each individual mode. The hybridised eigenmode frequencies

4 $${{{\Omega }}}_{\pm }={{{\Omega }}}_{{{m}}}-\frac{{{{\Omega }}}_{{{m}}}+{{\Delta }}}{2}\pm \sqrt{{g}_{{{y}}}^{2}+{\left(\frac{{{{\Omega }}}_{{{m}}}+{{\Delta }}}{2}\right)}^{2}}$$

experience an avoided crossing, the so-called NMS, which reaches a maximum of Ω_+_ − Ω_−_ = 2*g*_*y*_ at the optimal detuning Δ = − Ω_*m*_. The linewidth of the hybrid modes at this detuning is (*κ* + Γ_m_)/2. Therefore, Γ_m_ needs to be smaller or comparable to *κ* to resolve the NMS of 2*g*_*y*_.

As can be seen from Eq. (3), we control *g*_*y*_ through various parameters like the trap power *P*_t_, the particle position *y*_0_ and the polarisation angle *θ*. The optical coupling rate Γ_opt_ depends additionally on the trap detuning Δ and is maximised at Δ = − Ω_*m*_ to ${{{\Gamma }}}_{{\rm{opt}}}=4{g}_{{{y}}}^{2}/\kappa$ (refs. ^7^^[,12^). While *P*_t_ and Δ only influence the magnitude of the coupling strength, *y*_0_ and *θ* change also the nature of the coupling from 1D to potentially 3D^36^. For simplicity, we focus on varying Δ and *y*_0_ in the following measurements and keep *P*_t_, *θ* and Γ_m_ = 2*π* × 0.8 kHz, corresponding to *p* = 1.4 mBar, fixed (see “Experimental setup” section). The range of Δ is limited due to instabilities in the experiment.

### Observation of strong coupling

**Fig. 2:**
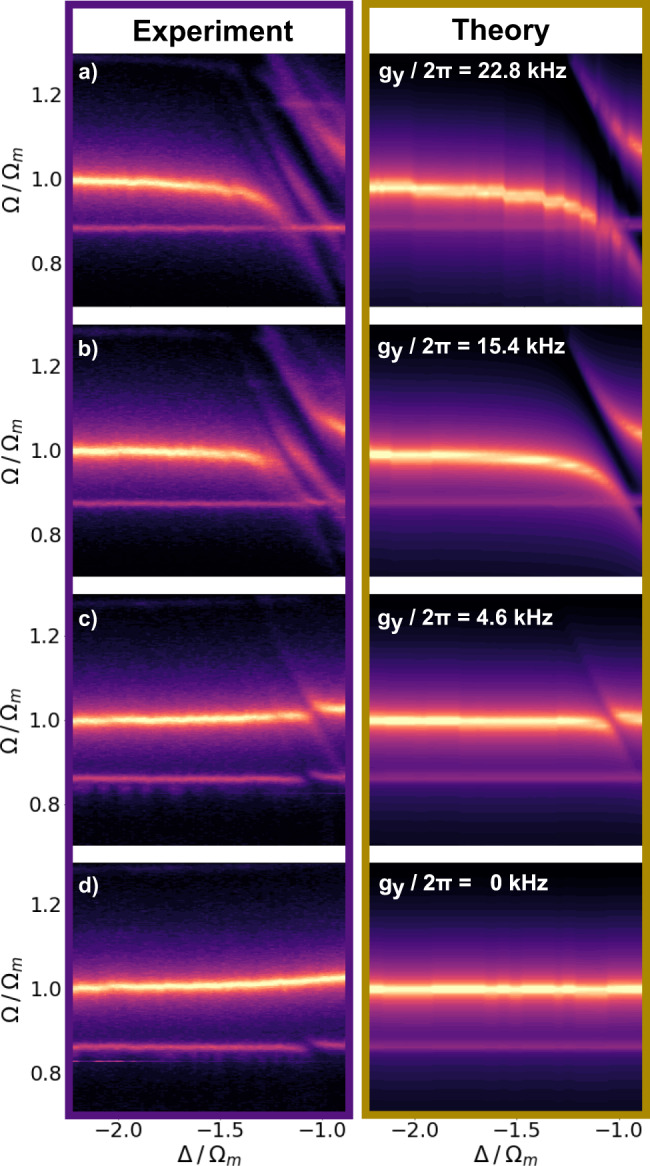
Normal mode splitting. Particleʹs position power spectral density PSD(Ω) versus Δ for different *y*_0_ and therefore various *g*_*y*_. Experimental data are displayed on the left, and theory on the right. The bare mechanical (optical) modes correspond to horizontal (diagonal) lines. **a** Maximum normal mode splitting of 2*g*_*y*_ is observed at Δ = − Ω_*m*_ yielding a value of *g*_*y*_ = 2*π* × 22.8 kHz = 2.3*κ*, where *y*_0_ ≈ *λ*_c_/4 is close to the intensity minimum (see Eq. (3)). **b** When the particle is moved by *δy*_0_ ≈ 0.12*λ*_c_, the coupling reduces to *g*_*y*_ = 2*π* × 15.4 kHz = 1.5*κ*. **c** Normal mode splitting is still visible at *δy*_0_ ≈ 0.2*λ*_c_, yielding *g*_*y*_ = 2*π* × 4.6 kHz = 0.46*κ*. **d** At the intensity maximum, corresponding to a shift of *δy*_0_ ≈ *λ*_c_/4 and *g*_*y*_ = 0 kHz, the normal mode splitting vanishes and we only see a shift of *δ*Ω_*m*_ ≈ 2*π* × 5 kHz in the mechanical frequency due to the increased intracavity photon number (see Supplementary Fig. 1). In general, we observe a good agreement between experimental data and theory. We attribute discrepancies to a second cross polarised cavity mode inducing a second normal mode splitting (for more details, see Supplementary Information).

**Fig. 3:**
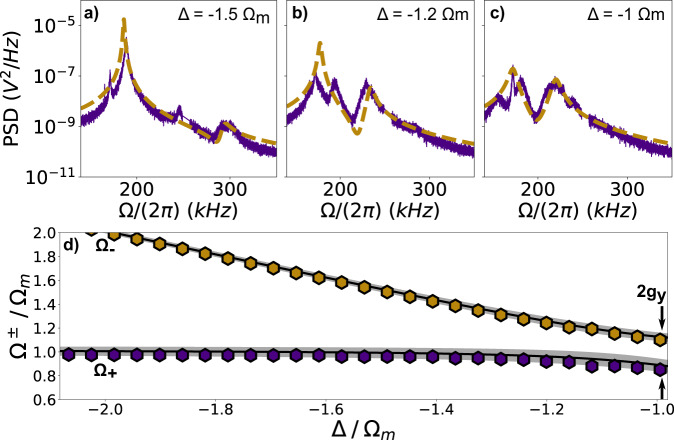
Power spectral density versus cavity detuning Δ. **a**–**c** Experiment (purple) and theory (yellow, dashed) fitted to Eq. (9) at Δ = −2*π* × 293 kHz ≈ −1.5Ω_*m*_ (**a**), Δ = −2*π* × 225 kHz ≈ −1.2Ω_*m*_ (**b**) and Δ = −2*π* × 205 kHz ≈ −Ω_*m*_ (**c**). The optomechanical coupling strength *g*_*y*_ grows with increasing Δ. Optical and mechanical modes start to hybridise clearly at Δ≥ −1.5Ω_*m*_. We attribute the discrepancy between data and theory to the second optical mode (see Supplementary Informtation). **d** Hybridised eigenmodes Ω_±_ versus Δ at the intensity minimum (*y*_0_ ≈ *λ*_c_/4). Maximum normal mode splitting of 2*g*_*y*_ with *g*_*y*_ = 2*π* × 22.8 kHz = 2.3*κ* occurs at Δ = −Ω_*m*_. The black line fits the data to Eq. (4), while the inner (outer) edges of the grey area correspond to a fit using solely to the upper (lower) branch Ω_−_ (Ω_+_).

**Fig. 4:**
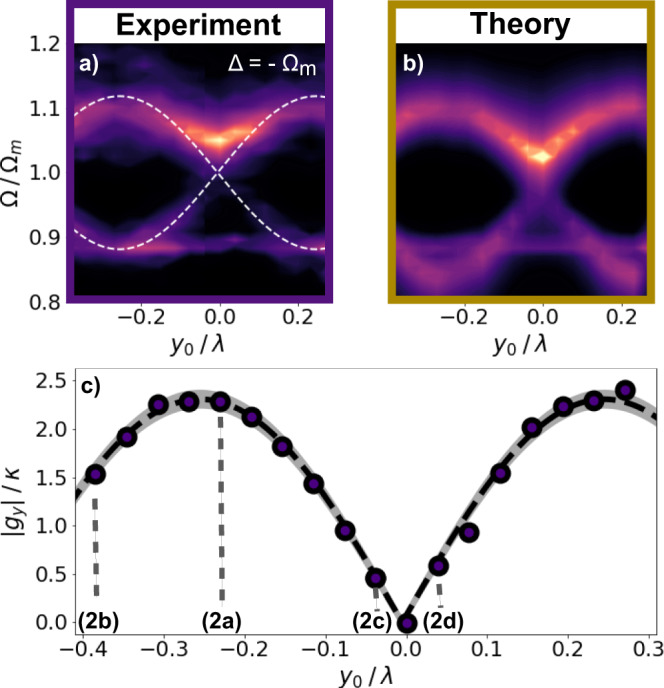
Normal mode splitting versus particle position *y*_0_. **a** Experiment and **b** theory according to Eq. (9). Particle position power spectral density PSD(Ω) at the optimal Δ = − 2*π* × 193 kHz ≈ −Ω_*m*_ along *y*_0_ is shown. The hybridised modes split by 2*g*_*y*_. The white dashed line displays Ω_±_/Ω_*m*_ = 1 ± *g*_*y*_/Ω_*m*_ where *g*_*y*_ follows Eq. (3). The mechanical mode at Ω/Ω_*m*_ ≈ 0.89 corresponds to the mechanical *x*−mode. The data and fit show very good agreement. **c** ∣*g*_*y*_∣ at Δ ≈ −Ω_*m*_ versus *y*_0_. Maximum and minimum coupling are separated by *δy*_0_ = *λ*_c_/4 as expected by Eq. (3). Black dashed line fits to the absolute value of Eq. (3) with a maximum ${g}_{{{y}}}^{\max }\approx -2.3\kappa$ and the grey shaded area corresponds to 3*σ*_std_ of the fit. The dotted lines indicate the positions used in Fig. 2.

Figure 2 left panel displays the experimental position power spectral density (PSD) versus Δ for different *y*_0_. Throughout the remaining part of the manuscript, we fit our PSD to Eq. (9), if not stated differently. From this fit, we can extract the hybridised modes Ω_±_ that are separated by 2*g*_*y*_. We cover a total distance of *δy*_0_ ≈ $\lambda$_c_/4 and change the optomechanical coupling strength, and therefore also the NMS, from (a) *g*_*y*_/2*π* = 22.8 kHz, (b) 15.4 kHz, (c) 4.6 kHz and (d) 0 kHz, exploring the entire range from strong coupling to zero coupling. The right panel shows the fit, which is in good agreement with the data. We observe two eigenmodes Ω_±_ with an exceptional NMS of 2*g*_*y*_ ≈ 4.6*κ* at Δ = − Ω_*m*_, corresponding to 20% of the bare mechanical eigenfrequency, once the system enters the SCR at *g* > *κ*/4 (ref. ^33^). For *g*_*y*_ = 0, we observe only the mechanical mode with slightly increased frequency Ω_*m*_ = 2*π* × 200 kHz due to the additional trapping potential supplied by the cavity field (see Fig. 2d). In Fig. 2a and b, we observe an additional NMS in the *y*-mode, which stems from a second cross polarised optical mode. Note that, throughout all our measurements (see Figs. 2–4), the second NMS is the largest source for discrepancies between experiment and theory (for more details, see Supplementary Information). We also attribute the NMS of the *x*-mode at Ω/Ω_*m*_ = 0.89 to the second optical mode as observed in Fig. 2d, since the *x*-mode should be decoupled from the first mode (*g*_*x*_ = 0 if *θ* = 0).

Figure 3a–c displays the particle’s position PSD at different Δ while it is located at the intensity minimum, corresponding to the position of maximum coupling *g*_*y*_ = 2.3*κ*, displayed in Fig. 2a. Our theory (yellow) captures the data (purple) well. In Fig. 3a, the optical mode and mechancial mode begin to hybridise into new eigenmodes at Δ = −1.5Ω_*m*_ which is confirmed by a second peak appearing at Ω ≈ 2*π* × 300 kHz. The hybridisation becomes stronger as Δ approaches the cavity resonance and the NMS is maximised at Δ ≈ −Ω_*m*_ as shown in Fig. 3c. The dependence of the new eigenmodes Ω_±_ on Δ is shown in Fig. 3d, displaying clearly the expected avoided crossing of 2*g*_*y*_. The solid line is a fit to Eq. (4). The edges of the shaded area represent the upper and lower limit of the fit, which we obtain by fitting only the upper branch (yellow) or the lower branch (purple), respectively.

As already discussed previously, our experiment allows to change the optomechanical coupling by changing various experimental parameters, which stands in contrast to many other experimental platforms. Figure 4 displays this flexibility to reach the SCR by demonstrating the position dependence of *g*_*y*_ at optimal detuning Δ ≈ −Ω_*m*_ extracted from the data Fig. 2a–d. The experimental and theoretical position PSDs versus *y*_0_ are depicted in Fig. 4a and b. The mode at Ω/Ω_*m*_ ≈ 0.89 corresponds to the decoupled *x*-mode. The dashed line highlights the theoretical frequency of the eigenmodes Ω_±_/Ω_*m*_ following Eq. (4). In both experiment and theory, we observe the expected sinusoidal behaviour predicted by Eq. (3). Figure 4c depicts ∣*g*_*y*_∣ = (Ω_+_ − Ω_−_)/2 (circles) extracted from Fig. 4a. The dashed line represents the fit to the absolute value of Eq. (3) yielding ${g}_{{{y}}}^{\exp }=2\pi \times (22.8\pm 0.2)$kHz which coincides well with the theoretical value of ${g}_{{{y}}}^{{\rm{th}}}=2\pi \times 22.6$ kHz. The measured period coincides with the expected period of $\lambda$_c_/4. The shaded area corresponds to 3*σ*_std_ of the fit.

## Discussion

As a figure of merit to assess the potential of our system for quantum applications, we use the quantum cooperativity, which yields here ${C}_{{\rm{CS}}}={(2{g}_{{{y}}}^{\max })}^{2}/(\kappa {{{\Gamma }}}_{{\rm{m}}}({n}_{{\rm{th}}}+1))=8\times 1{0}^{-6}$ at a pressure *p* = 1.4 mbar and promises a value as large as *C*_CS_ ≈ 36 at *p* = 3 × 10^−7^ mbar, since Γ_m_ ∝ *p*. At this low pressure, the photon recoil heating rate Γ_rec_^41^ equals our mechanical decoherence rate Γ_m_(*n*_th_ + 1)), and therefore halves the reachable *C*_CS_. The maximum *C*_CS_ is ultimately limited by Γ_rec_, regardless if we reduce the pressure even further. Nevertheless, this predicted value of *C*_CS_ is many orders of magnitude larger than what has been achieved in levitation setups by actively driving the cavity^7,24^ and larger than achieved in ref. ^9^. More importantly it enables coherent quantum control at *g* ≫ *κ*, Γ_m_ ⋅ *n*_th_ at pressure levels *p* ≤ 10^−6^ mbar, a pressure regime commonly demonstrated in numerous levitation experiments^7,9,41^.

Furthermore, our experimental parameters promise the possibility of motional ground state cooling in our system^9^, which in combination with coherent quantum control enables us to fully enter the quantum regime with levitated systems and to create non-classical states of motion and superposition states of macroscopic objects in free fall experiments^10,11^ in the future.

## Methods

### Experimental setup

**Fig. 5:**
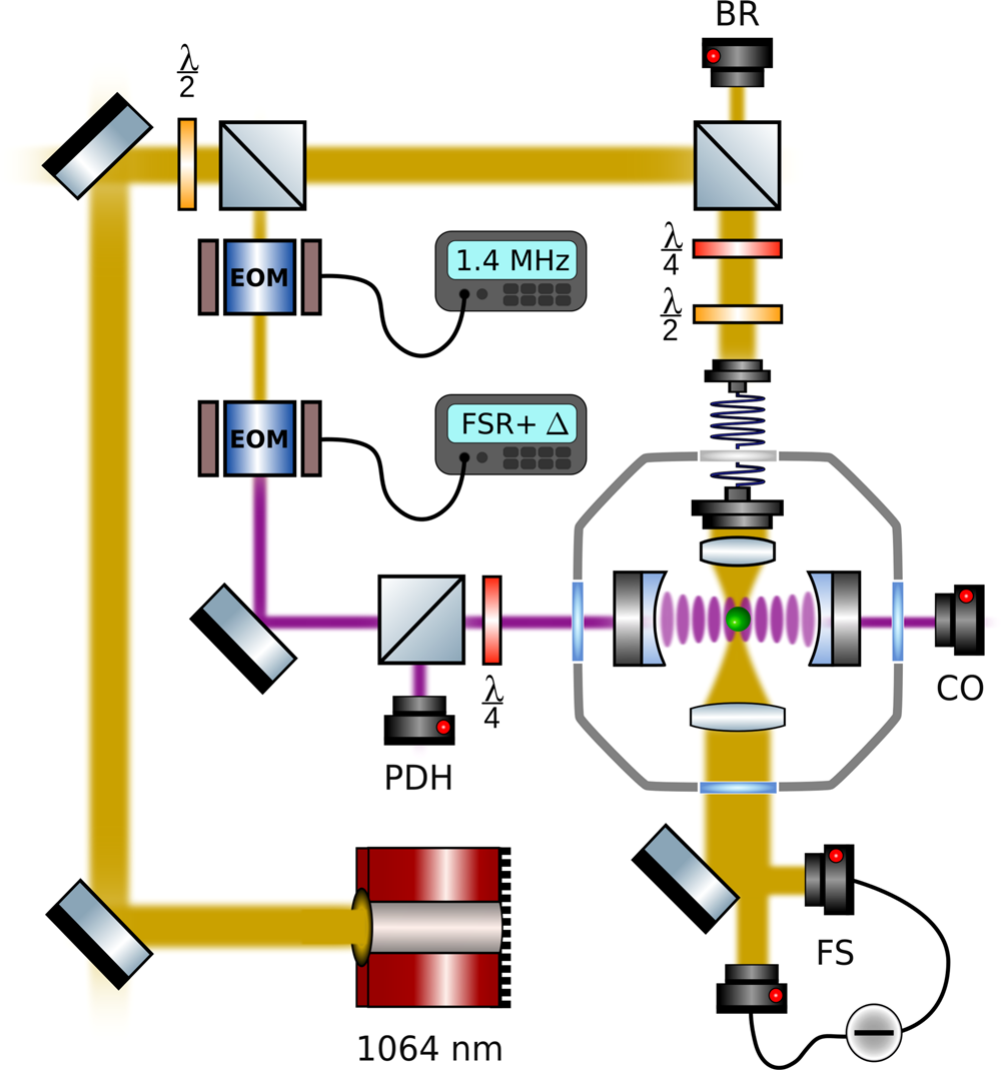
Extended experimental setup. A 1064 nm Mephisto laser (yellow) traps a silica particle of *d* = 177 nm inside a high finesse cavity (purple). The trap light is locked at a variable detuning Δ + FSR from the cavity resonance via the Pound-Drever-Hall technique by detecting the error signal on a photodiode (PDH). The particle motion is detected in backreflection (BR) and balanced forward detection (FS). The intracavity field is estimated from the transmitted power detected on a photodiode (CO).

The experimental setup is displayed in Fig. 5. A silica nanoparticle is loaded at ambient pressure into a long range single beam trap and transferred to a more stable, short range optical tweezers trap^42^ (with wavelength $\lambda$_t_ = 1064 nm, power *P* ≃ 150 mW, focusing lens NA = 0.8) inside a vacuum chamber. Due to the tight focusing, the nanoparticle non-degenerate eigenfrequencies are Ω_*x*,*y*,*z*_ = 2*π* × (172 kHz, 197 kHz, 56 kHz), respectively. The optical tweezers are mounted on a 3D nanometre resolution piezo system allowing for precise 3D positioning inside a high finesse Fabry-Perot cavity (with cavity finesse *F* = 540,000, free spectral range FSR = 2*π* × 5.4 GHz).

In order to control the detuning Δ = *ω*_t_ − *ω*_c_ between the cavity resonance *ω*_c_ and the trap field *ω*_t_, we use a weak cavity field for locking the cavity via the Pound-Drever-Hall technique (PDH) on the TEM01 mode minimising additional heating effects through the photon recoil heating of the cavity lock field. The PDH errorsignal acts on the internal laser piezo and an external AOM (not shown). We separate lock and trap light in frequency space by one free spectral range (FSR) such that the total detuning between lock and trap yields *ω*_t_ = *ω* − FSR − Δ. The variable EOM modulation *FSR* + Δ is provided by a signal generator. The intracavity power can be deduced from the transmitted cooling light observed on a photodiode behind the cavity (CO).

All particle information shown is gained in forward balanced detection interfering the scattered light field and the non-interacting part of the trap beam as shown in Fig. 5. The highly divergent trap light is collected using a lens (NA = 0.8). We use three balanced detectors (FS) to monitor the oscillation of the particle in all three degrees of freedom.

The data time traces are acquired at 1 MHz acquisition rate. Each particle position PSD is obtained by averaging over *N*  = 25 samples of which each one is calculated from individual 40 ms time traces, corresponding to a total measurement time of *t* = 1 s.

We keep the pressure stable at *p* = 1.4 mbar. The thermal bath couples as

5 $${{{\Gamma }}}_{{\rm{m}}}=\frac{{k}_{{\mathrm{B}}}T}{\hslash {Q}_{{\rm{m}}}{n}_{{\rm{th}}}}=15.8\frac{{R}^{2}p}{m{v}_{{\rm{gas}}}}$$

where *Q*_m_ = Ω_*m*_/Γ_m_ is the mechanical quality factor, ${n}_{{\rm{th}}}=\frac{{k}_{{\mathrm{B}}}T}{\hslash {{{\Omega }}}_{{{m}}}}$ the thermal occupation number, *R* the particle radius, *p* the surrounding gas pressure and ${v}_{{\rm{gas}}}=\sqrt{3{k}_{{\mathrm{B}}}T/{m}_{{\rm{gas}}}}$.

In the measurements presented, we cool our particle’s centre of mass motion to *T* = 235, corresponding to a reduction of the phonon occupation by roughly 20%. The theoretically expected heating rate due to the residual gas accounts fully for the experimentally observed heating rate.

### Interaction Hamiltonian and power spectral densities

Following ref. ^36^, the relevant contributions to the CS interaction Hamiltonian for *θ* = 0 are given by

6 $$\frac{{\hat{{\rm{H}}}}_{{\rm{CS}}}}{\hslash }=-{g}_{{{y}}}({\hat{a}}^{\dagger }+\hat{a})({\hat{{b}_{y}}}^{\dagger }+\hat{{b}_{y}})\\ -{g}_{{{z}}}({\hat{a}}^{\dagger }-\hat{a})({\hat{{b}_{z}}}^{\dagger }+\hat{{b}_{z}})$$

7 $$\frac{{\hat{{\rm{H}}}}_{{\rm{DR}}}}{\hslash }=-{g}_{{{y}}}^{{\rm{dr}}}\,{\hat{a}}^{\dagger }\hat{a}\,({\hat{{b}_{y}}}^{\dagger }+\hat{{b}_{y}})$$

8 $$\frac{{\hat{{\rm{H}}}}_{{\rm{CAV}}}}{\hslash }=-\frac{{G}_{\perp }}{2}({\hat{a}}^{\dagger }+\hat{a})\cos \phi$$

The single photon optomechanical coupling strength achieved by actively driving the cavity is ${g}_{{{y}}}^{{\rm{dr}}}=\frac{\alpha {\omega }_{{\rm{c}}}}{2{\epsilon }_{0}{V}_{{\rm{c}}}}{k}_{{\rm{c}}}{y}_{{\rm{zpf}}}\sin (2\phi )=2\pi \times 0.05\;{\rm{Hz}}\sin (2\phi )$. This value is enhanced by the intracavity photon number *n*_cav_ = 1.6 × 10^8^, inferred from the transmitted cavity power *P*_out_. At optimal conditions, we achieve ${g}_{{{y}}}^{{\rm{rp}}}\sqrt{{n}_{{\rm{c}}}}=2\pi \times 0.6$ kHz. Thus, the optomechanical coupling strength is about 40 times larger for CS, since the photons contributing to the CS interaction are confined in a much smaller volume due to the much smaller trap waist ${{{w}}}_{{\mathrm{t}}}\times {{{w}}}_{{\mathrm{c}}}\ll {{{w}}}_{{\mathrm{c}}}^{2}$.

The mechanical susceptibility *χ* is given as^33^

9 $$| \chi ({{\Omega }}){| }^{2}=\frac{1}{{m}^{2}[{({{{\Omega }}}_{{{m}}}^{2}+2{{\Omega }}\delta {{{\Omega }}}_{{{m}}}({{\Omega }})-{{{\Omega }}}^{2})}^{2}+{({{\Omega }}{{{\Gamma }}}_{{\rm{eff}}}({{\Omega }}))}^{2}]}$$

10 $${{{\Gamma }}}_{{\rm{eff}}}({{\Omega }})={{{\Gamma }}}_{{\rm{m}}}+{{{\Gamma }}}_{{\rm{opt}}}({{\Omega }})$$

11 $$\delta {{{\Omega }}}_{{{m}}}({{\Omega }})={g}_{{{y}}}^{2}\frac{{{{\Omega }}}_{{{m}}}}{{{\Omega }}}\left[\frac{{{\Delta }}+{{\Omega }}}{{({{\Delta }}+{{\Omega }})}^{2}+{\kappa }^{2}/4}+\frac{{{\Delta }}-{{\Omega }}}{{({{\Delta }}-{{\Omega }})}^{2}+{\kappa }^{2}/4}\right]$$

12 $${{{\Gamma }}}_{{\rm{opt}}}({{\Omega }})={g}_{{{y}}}^{2}\frac{{{{\Omega }}}_{{{m}}}}{{{\Omega }}}\left[\frac{\kappa }{{({{\Delta }}+{{\Omega }})}^{2}+{\kappa }^{2}/4}-\frac{\kappa }{{({{\Delta }}-{{\Omega }})}^{2}+{\kappa }^{2}/4}\right]$$

with the effective (optical) damping Γ_eff_ (Γ_opt_) and the optomechanical spring effect *δ*Ω_*m*_. We fit the three mechanical modes Ω_*x*,*y*,*z*_ to Eq. (9) where *g*_*y*_, *κ*, Γ_m_ and the relative mode amplitudes are chosen as free fit parameters.

## Supplementary information

Supplementary Information

## Data Availability

The data that support the findings of this study are available from the corresponding author upon request.
