# Supplementary material for: Strong optomechanical coupling at room temperature by coherent scattering
Source: Nat Commun. 2021 Jan 12;12:276. doi: 10.1038/s41467-020-20419-2 (PMC7803762; doi:10.1038/s41467-020-20419-2)
Supplement: Supplementary file 1 — Supplementary Information [file 41467_2020_20419_MOESM1_ESM.pdf]

Supplementary Information for: Strong Optomechanical Coupling at Room Temperature by Coherent Scattering

A. de los Ríos Sommer et al.

Supplementary Note 1: Multimode Coupling

In Fig.2(**a**) and (**b**) an additional NMS at $\Delta = -\Omega_m - 2\pi \times 34$kHz is observed. We attribute this to a second cross polarised cavity mode. The frequency difference in between these optical modes has been measured independently to $\delta\Delta = 2\pi \times 34$kHz, which corresponds exactly to the shift observed in the data here. The presence of the second optical mode is experimentally unavoidable due to the high finesse character of the cavity.
In [1] the contribution of photons polarised along z was neglected ($G_z = 0$), since the trap has nominally no polarisation component along this axis. Due to tight focusing as done here, this assumption breaks down and $G_z \neq 0$.
Our claim is supported by the observation of an unexpected NMS in the x-mode ($g_x \neq 0$) at $\Omega/\Omega_m = 0.89$ as observed in Fig.2, despite the expected $g_x = 0$ for $\theta = 0$. We exclude that this effect stems from polarisation contributions $\epsilon_y \neq 0$ or non-linear contributions [2] due to the observed magnitude of g_x and Δ.
The presence of a second optical mode with $G_z \neq 0$ gives rise to an optomechanical coupling, so far neglected in experiments and theory. The presence of several mechanical and optical modes simultaneously interacting through the cavity enables the study of interesting multimode effects in the future like dark modes [3], mechanical synchronisation effects [4], and entanglement [5, 6].

Supplementary Note 2: Cavity Readout

The intracavity field is estimated from the transmitted cavity light (CO in Fig.1) and the measured absorption, transmission and reflection of the cavity mirrors. The intracavity power versus Δ is depicted in Supplementary Fig.1(a). When y_0 is close to the intensity maximum ($g_y = 0$), the transmitted power is maximal and we see the expected increase in intensity with increasing Δ following the usual Lorentzian lineshape. In contrast, at the intensity minimum ($g_y = 2.3\kappa$), the transmission vanishes independently of Δ. Supplementary Fig.1(b) displays the position dependence of the intracavity power along the cavity axis for optimal detuning $\Delta = -\Omega_m$. The intracavity power follows a sinusoidal shape, where the intensity maximum coincides with positions where $g_y = 0$ and the intensity minimum with $g_y = 2.3$ respectively.

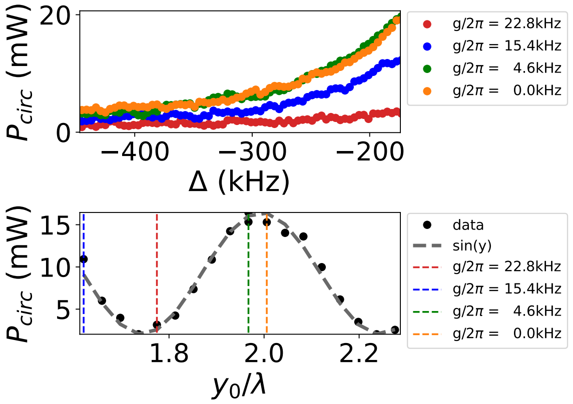

Supplementary Figure 1: **Transmitted Cavity Power** (**a**) versus Δ and (**b**) versus y_0

Supplementary Note 3: Particle Solution Preparation

With the help of a commercial nebulizer we produce microsized droplets of an ethanol-nanoparticle solution. We work with nanoparticles of diameter $d = 177$nm. In order to avoid the creation of clusters, we mix a solution of 3ml Ethanol with 3μl nanoparticle-water-solution where each droplet has a probability of $\rho \lesssim 1$ to contain a nanoparticle. This solution is sprayed through a metal funnel to direct the particle flow towards the trapping region and to possibly slow down the droplets to increase the trapping probability. We achieve a trapping event around every three seconds of which > 50% can be used for experiments. Cluster trapping events can be identified by measuring the brightness of the trapped object on a CCD camera in real time.

Supplementary References

[1] Gonzalez-Ballestero, C. *et al.* Theory for Cavity Cooling of Levitated Nanoparticles via Coherent Scattering: Master Equation Approach. *Physical Review A* **100**, 013805 (2019).

[2] Toroš, M. & Monteiro, T. S. Quantum sensing and cooling in three-dimensional levitated cavity optomechanics. *Physical Review Research* **2**, 1–4 (2020).

[3] Dong, C., Fiore, V., Kuzyk, M. C. & Wang, H. Optomechanical Dark Mode. *Science* **338**, 1609–1613 (2012).

[4] Sheng, J., Wei, X., Yang, C. & Wu, H. Self-Organized Synchronization of Phonon Lasers. *Physical Review Letters* **124**, 53604 (2020).

[5] Riedinger, R. *et al.* Remote quantum entanglement between two micromechanical oscillators. *Nature* **556**, 473–477 (2018).

[6] Chen, J., Rossi, M., Mason, D. & Schliesser, A. Entanglement of propagating optical modes via a mechanical interface. *Nature Communications* **11** (2020).
